# The Impacts of Climate Change Mitigation Strategies on Animal Welfare

**DOI:** 10.3390/ani5020361

**Published:** 2015-05-20

**Authors:** Sara Shields, Geoffrey Orme-Evans

**Affiliations:** Humane Society International, 2100 L Street NW, Washington, DC 20037, USA; E-Mail: gevans@hsi.org

**Keywords:** animal welfare, climate change, mitigation strategies

## Abstract

**Simple Summary:**

Climate change is probably the most important environmental issue of our time. Raising animals for food contributes to the production of greenhouse gases implicated in the global warming that is causing climate change. To combat this ecological disaster, a number of mitigation strategies involving changes to agricultural practices have been proposed. However, some of these changes will impact the welfare of farmed animals. This paper reviews selected climate change mitigation strategies and explains how different approaches could have negative or positive effects.

**Abstract:**

The objective of this review is to point out that the global dialog on reducing greenhouse gas emissions in animal agriculture has, thus far, not adequately considered animal welfare in proposed climate change mitigation strategies. Many suggested approaches for reducing emissions, most of which could generally be described as calls for the intensification of production, can have substantial effects on the animals. Given the growing world-wide awareness and concern for animal welfare, many of these approaches are not socially sustainable. This review identifies the main emission abatement strategies in the climate change literature that would negatively affect animal welfare and details the associated problems. Alternative strategies are also identified as possible solutions for animal welfare and climate change, and it is suggested that more attention be focused on these types of options when allocating resources, researching mitigation strategies, and making policy decisions on reducing emissions from animal agriculture.

## 1. Introduction

Climate change is arguably the most important environmental issue of our time. With severe and widespread destructive effects, warming of the planet threatens ecological systems, peoples’ livelihoods, and species survival [1]. Animal agriculture is an important source of greenhouse gas (GHG) emissions and has been implicated as a serious contributor to climate change [2]. To reduce the contribution of emissions attributable to animal agriculture, a number of mitigation strategies involving changes to farming practices have been proposed. Although this is an important and timely goal, and many of the proposed solutions seem reasonable on the surface, mitigation strategies can have complex effects on people and animals in practice. While there has been occasional mention [3], in the global discussion on climate change there has generally been a dearth of attention paid to the animal welfare impacts of the proposed abatement options [4,5], and some of the suggested livestock management approaches would have severe and wide-ranging impacts on the animals.

At the same time, in other arenas there is a growing international social movement afoot aimed at addressing animal welfare, the physical and psychological state of animals [6]. There are many animal welfare issues associated with commercial farming practices, especially in industrial agricultural production systems, where animals are often confined indoors at high stocking densities. The conditions in which the animals are kept are a matter of serious deliberation in legislative, corporate, investment and trade organizations, among many others around the world. Overarching calls to increase animal productivity, by, for example, pushing animals (either genetically through selective breeding, with hormone drugs, or through feed changes) to produce more milk or meat, or to produce more offspring faster, can also be considered in light of potential health and welfare consequences.

This paper argues that the potential impact of climate change mitigation strategies on animal welfare should be acknowledged and considered in the ongoing debate and discussion centered on climate change approaches and climate change research, with the aim of maximizing co-benefits of sustainability while avoiding negative tradeoffs. Specific mitigation strategies are discussed and their potential impacts on the animals are assessed. As examples of alternative pathways forward, some mitigation approaches that have no effect on animal welfare or even a positive effect are also reviewed. Given the scope and severity of the impacts on animals, and the increasing global support for more welfare-friendly practices, animal welfare concerns should become a more prominent part of the dialog and policy decisions on climate change mitigation practices in animal agriculture.

### Background on Climate Change and Mitigation Strategies

The Earth’s surface has been successively warmer over each of the last three decades compared to any preceding decade since 1850 [7]. Human activities are responsible (with a 95%–100% probability) for the recent global warming and the marked increase in global atmospheric concentrations of carbon dioxide (CO_2_), methane (CH_4_) and nitrous oxide (N_2_O) to above pre-industrial values [8]. By sector, greenhouse gas pollution originates primarily from industry; agriculture, forestry and other land use (AFOLU); buildings; and transport [9]. Separated out, animal agriculture emits a significant portion, estimated from 7% to 18% of anthropogenic greenhouse gas emissions [10].

Livestock contribute both directly and indirectly to climate change. Enteric fermentation and manure associated emissions are direct, while production and transport of feed (including the fossil fuels used in manufacturing chemical fertilizers) and land use changes (such as conversion of forest to pasture and crop land) contribute indirectly [10]. About 44% of the emissions generated by livestock are CH_4_, which is released during enteric fermentation (eructation in ruminants) and emitted from manure decomposition; 27% are in the form of CO_2_ emitted during the production and transport of animal products and feed, and 29% are N_2_O attributable to manure and fertilizer [2]. There is a clear need to address the contribution that farming animals makes to global warming.

To combat climate change caused by animal agriculture, a number of mitigation strategies have been proposed, as well as policy options to implement such strategies [11]. A theme that runs through this literature is greater efficiency of production, with the aim to achieve more output for a given level of resources used. As explained in the pivotal 2006 United Nations, Food and Agriculture (FAO) report, *Livestock’s Long Shadow*:

The most promising approach for reducing methane emissions from livestock is by improving the productivity and efficiency of livestock production, through better nutrition and genetics. Greater efficiency means that a larger portion of the energy in the animals’ feed is directed toward the creation of useful products (milk, meat, draught power), so that methane emissions per unit product are reduced. The trend towards high performing animals and towards monogastrics and poultry in particular, are valuable in this context as they reduce methane per unit of product. The increase in production efficiency also leads to a reduction in the size of the herd required to produce a given level of product. Because many developing countries are striving to increase production from ruminant animals (primarily milk and meat), improvements in production efficiency are urgently needed for these goals to be realized without increasing herd sizes and corresponding methane emissions.

[12] (*pp. 119–120*)

However, by far, the most substantial emissions reductions can come through adaptations in current systems rather than requiring a shift to industrialized systems [2]. This is important from an animal welfare perspective. While there can be negative animal welfare impacts in all production systems, industrialized production has inherently problematic effects on billions of animals globally. Thus, it is important that there is significant alignment in both animal welfare and climate goals. Some ways in which they are less compatible are discussed in Section 2.

## 2. Mitigation Strategies and Possible Negative Animal Welfare Effects

### 2.1. Feed Changes

Given that feed production accounts for about 47% of livestock emissions [2] (p. 18), it is a key target for mitigation. However, there are a number of welfare concerns with some of the current proposals to reduce emissions using feed alterations.

#### 2.1.1. Grain Finishing Feedlot Cattle

One strategy for mitigating methane emissions with feed changes is by including more concentrates in addition to, or in place of, a proportion of dietary forage. Although emissions per animal may rise as productivity improves, there is generally a reduction in the greenhouse gases released on a per kg-product produced basis [10,13]. In intensive production systems, grain feeding of livestock often entails removing cattle raised for beef from pastures to provide the finishing ration in a feedlot. This is a common system in the United States and is being emulated in other parts of the world, such as Latin America. In Brazil, the practice of finishing cattle in feedlots increased 50% (from two to three million animals) from 2003 to 2010 [14].

The digestive system of cattle is best adapted to roughage provided by grass-based diets [15], and the natural behavior of cattle is to graze throughout the day. However, the typical routine in feedlots is to gradually reduce the proportion of forage in the ration over time, eventually reaching diets that can be as high as 90% grain [16]. In enlarging Brazilian feedlots, the expected trend is a decrease in the amount of roughage in feedlot diets, with an already observed reduction from 29% in 2009 to 21% in 2011 [17].

In cattle feeding systems with high ratios of concentrate to forages, digestive problems are common. The starches in this concentrated diet are quickly digested and fermentation acids can build up disrupting the normal function of the rumen [16]. Abnormal rumen function and digestive disorders can lead to acidosis, bloat, and if persistent, liver abscesses, and even the foot disorder laminitis [15,18,19]. Maintaining normal rumen function on these diets is a constant challenge for the feedlot industry; one quarter of cattle mortality in feedlots can be attributed to digestive disorders [20].

For both acidosis and bloat, increasing the amount of forage in the diet can stimulate chewing and promote saliva production, which have a buffering effect, and thus reduce the likelihood of a sinking pH level and destabilization of the microbial population in the rumen [16,21].

Finishing calves in a feedlot usually involves transporting them from their natal pastures. In the United States, nearly two-thirds (63%) of cattle and calves raised for beef are sold through an auction barn after leaving the farm [22], thus, many calves are moved twice before arrival at a feedlot.

Transport takes a physical and psychological toll on animals. Stressors include unfamiliar surroundings, novelty, noise, vibration, social regrouping, loading and unloading, and feed and water deprivation [23,24,25], although many factors, such as preconditioning [26] and location in the vehicle during the journey [27] can alter the effect. The stress of transport can lead to immunosuppression and an increase in disease susceptibility, including the ubiquitous Bovine Respiratory Disease (RBD) problem [25,28,29].

#### 2.1.2. Concentrated Diets for Pigs

In cases where intensification of animal agriculture is accompanied by better access to resources, then there may be benefits for welfare in terms of providing more nutritionally complete rations. However, pigs are also often fed highly concentrated grain-based diets in intensive production. Concentrated diets may be fed to pigs in a finely ground or pelleted ration and are expected to reduce GHG emissions due to their high digestibility. For example, in a case study of intensive pig production in East and South East Asia, the FAO explored changes to swine diets as a potential mitigation strategy, including increased digestibility of feed. In intermediate (not fully industrialized) systems, by changing the feed along with other management changes, emissions could be reduced by 32% to 38% [2].

Pigs’ stomachs are biologically designed for numerous, small meals throughout the day of high fiber feedstuffs [30]. However, in intensive production, they have little access to roughage. Feed processing improves feed-to-gain efficiency, but it is also associated with intestinal problems. These low fiber diets can impact mucosa integrity leaving pigs prone to gastric ulcers in the pars esophageal region of the stomach [31]. This is a problem worldwide. Reports vary, with incidence of pigs showing distinct signs of ulceration between farming operations ranging from zero to nearly 90% [32,33,34,35,36]. For pigs in the growing/finishing stages of production, ulceration is a common cause of mortality [37]. The production systems in which most pigs are kept appear to have a large impact on the incidence of these ulcers, as pigs with access to straw, sawdust, or outdoor paddocks have fewer ulcers than those confined on bare, solid or slatted concrete floors [32,38,39].

#### 2.1.3. Feed Additives

Feed additives can be used to inhibit CH_4_ production in a variety of species. However, some dietary additives that are being tested have potential animal health concerns. For example, *in vitro* studies have shown that fumarate can work as an alternative to CH_4_ as a sink for H_2_ in the rumen, but feeding fumarate in its free acid form can lower rumen pH and inhibit fiber digestion, while feeding it in salt form can induce toxicity. Effective doses may well exceed current dietary recommendations [40]. Especially in low protein diets, nitrates are also potential CH_4_ mitigation agents. However, the intermediate product from nitrate metabolism, nitrite, is toxic, and so feeding it requires a gradual acclimation to a high dietary intake in order to avoid nitrite toxicity [10,41]. Another proposed supplement, sulfate, has been linked to sulphur-induced polioencephalomalacia, a condition in US feedlot cattle associated with excessive production of H_2_S in the rumen from diets high in distillers grains—co-products from the ethanol industry, which are high in sulfate [10,42]. The FAO cautions that more research is needed [10]. Mitigation strategies should steer clear of feeding substances that might endanger the animals, either outright or in the absence of highly skilled management.

### 2.2. Genetic Selection

In general, as animals become more productive, greater feed intake is needed to support the energy requirements associated with growth or lactation, but the proportion of total energy requirements required for maintenance decreases [43]. Thus the feed input per unit of product produced is reduced. Further, with increases in productivity, fewer animals are needed to reach a given level of output [43]. In the climate change mitigation literature, this concept is often exemplified with dairy cows. For example, in 1944, the diverse breeds comprising the US dairy cow population produced an annual milk yield of 2074 kg per cow, but by 2007 the genetic pool had shrunk, and Holsteins were the dominant breed, while milk production more than quadrupled to 9193 kg per cow. In 1944 there were 25.6 million dairy cows in the United States with an annual production of 53.0 billion kg of milk, while in 2007 the total US dairy herd had declined to 9.2 million cows, but they produced 84.2 billion kg of milk. Because of this per cow increase in production, the total carbon footprint for the US dairy industry in 1944 was 194 million tons of CO_2_-equivalent (CO_2_-eq.), while in 2007 the industry’s carbon footprint was only 114 million tons [44]. Much of this individual increase in productivity can be attributed to genetic selection, although dietary changes also contributed.

Swine and poultry produce relatively small amounts of enteric CH_4_, but their manure can be a significant source of GHG production. Therefore, maintaining or improving feed conversion efficiency in these species, and subsequently reducing the volume of manure produced for a given level of output, is considered a major strategy for mitigating CH_4_ and N_2_O [45]. However, there is evidence across species that overemphasis on productivity in genetic selection can lead to unintended negative effects on animal welfare.

#### 2.2.1. Effects of Breeding for Yield on Dairy Cow Welfare

While selective breeding has been successful in producing cows that yield more milk with less feed, this change is not without costs. Detrimental, unintended side-effects on the health of dairy cows have accompanied the physiological demands of greater productivity [46].

Following the birth of her calf, a dairy cow enters a state of negative energy balance. Genetic selection for increased milk yield increases feed intake, but high yielding cows can experience greater negative energy balance [47] than cows producing less, so they are vulnerable to health problems. There is a strong correlation between maximum metabolic load and laminitis [48], lameness and ketosis [49,50]. Failure to meet the nutritional demands of highly productive dairy cows can also lead to an array of additional metabolic diseases including fatty liver disease, ketosis and periparturient hypocalcemic paresis (milk fever) [51]. 

Mastitis, a bacterial infection of the udder, is the most commonly reported health problem in the US dairy industry, affecting 16.5% of cows [52]. The udder and teats of mastitic cows are sensitive to the touch, and in severe cases, clinical signs include swelling and pain sometimes accompanied by fever and depression. It is one of the leading reasons for culling of cows [52]. Although there are many risk factors for mastitis, such as lack of cleanliness in cow housing areas, research has consistently shown an association between mastitis and the amount of milk produced. For example, a study of over 3000 cows in 85 trials in eight different countries found that even with similar management conditions, higher yielding cows contracted more mastitis than lower yielding cows [53]. Many studies have reported that milk yield and mastitis are genetically correlated [54,55,56,57,58,59].

Further, high genetic merit dairy cows require high energy feeds to meet the substantial energy demands of early lactation. They often receive a high proportion of cereal grains, and one model found that cows selected for greater milk fat and protein fed a 50% grain diet had lower emissions (1.05 kg CO_2_-eq. per kilogram of energy-corrected milk), than cows with a 25% grain diet and summer grazing (1.19 kg per kilogram of energy-corrected milk) [60]. However, dairy cows are prone to digestive disorders such as sub-acute ruminal acidosis (SARA) [61,62]. Diets that are rich in grains alter rumen metabolic balance and can induce an explosive proliferation of *Streptococcus bovis* and *Lactobacillus* bacteria species. [63]. The resulting rumen acidosis eventually leads to erosion and ulceration of the ruminal epithelium and chronic symptoms including lethargy, reduced feed intake, diarrhea, and discomfort among others.

#### 2.2.2. Effects of Breeding for Productivity on Swine Welfare

Increases in the productivity of swine, largely achieved through genetic selection, have been cited as climate change mitigation progress [10] (p. 100–101). For example, a 2013 FAO report cited data provided by the US National Pork Board. Between 1959 and 2009, there was a 29% increase in the number of hogs marketed in the United States, yet the size of the breeding herd has been reduced by 39%. This is due in part to the steady increase in litter size from 7.10 in 1974 to 9.97 piglets in 2011 and the concomitant change in the amount of pork produced from a single breeding animal from 775 to 1828 kg. Feed efficiency also increased by 33%. This has reduced the carbon footprint per 454 kg of hot dressed carcass weight produced by 35% [10,45]. However, the original source for this calculation [64] does not appear to be peer-reviewed, and total emissions in the US pork industry have increased overall with greater pork production. Despite this, the suggestion is that the type of throughput achieved in the United States, if adopted more widely in the developing world, could be an approach to climate change mitigation. However, some of the key unintended animal welfare consequences that may accompany such a change include low piglet viability and severe feed deprivation of breeding sows in order to prevent them from becoming obese.

Selectively breeding for increased litter size reduces the proportion of surviving piglets [65,66]. Herpin *et al.* (1993) postulated that genetic changes have altered body fat metabolism, body composition, and hormonal state, resulting in lean tissue growth that makes piglets heavier but less mature at birth compared to lines that have not been so intensely selected for production traits. This reduces piglet survival rate [67], which is an intractable problem in the pork industry. Selection for leanness may have also inadvertently decreased the nutritional quality of sows’ milk [68], in turn further affecting survival of the piglets.

Selectively bred for productivity, growing/finishing pigs in the United States are fed to satiety to meet their full genetic potential, but breeding animals must often be feed restricted to prevent excessive weight gain and fat deposition. Confined sows are typically fed just half the amount they would choose to consume if fed *ad libitum*. This leads to a chronic hunger problem including persistent, unfulfilled feeding motivation [69,70,71]. Short feeding times and lack of an outlet for natural foraging behavior are underlying causes of abnormal stereotypic behavior, discussed below in Section 2.4.1.

#### 2.2.3. Effects of Breeding for Productivity on the Welfare of Chickens

As with swine, genetically linked productivity improvements in poultry have also been tied to climate change benefits through feed conversion efficiency [10] and the extremely rapid growth rate of modern broiler chickens (raised for meat). Broilers now grow from hatch to market weight in five weeks or less. Annual growth rates have increased 3.3% over the past 50 years, and broiler chickens now grow more than four times faster than they did in 1957 [72]. Using a life-cycle assessment model, one study estimated that genetic changes in average U.K. broiler chickens have achieved a 1% reduction per year in greenhouse gases over the last 20 years [73].

These animals now grow so fast that muscle outpaces bone development, leading to metabolic bone disease. As a result, broiler chickens often suffer from leg deformities and lameness [74,75,76], including tibial dyschondroplasia, angular bone deformities, spondylolisthesis, epiphyseitis, and in severe cases, rupture of the gastrocnemius tendon. Studies are consistent in showing that approximately 26%–30% of broiler chickens by 40–42 days of age suffer from gait defects severe enough to impair walking ability [76,77,78]. Additional research strongly suggests that while conformational differences account for some gait differences [79], birds at this level of lameness are probably in pain [80,81,82].

Not only do rapidly growing broiler chickens suffer from musculoskeletal diseases, but they are also afflicted by metabolic disorders. Ascites is a condition in which rapidly growing broiler chickens do not have the heart and lung capacity needed to distribute oxygen throughout the body and is a leading cause of mortality as the birds reach market weight [83]. Sudden Death Syndrome (SDS) is associated with acute heart failure caused by dysrhythmias. Young birds die from SDS after sudden convulsions and wing-beating, and are frequently found lying on their backs [84]. The condition has been recognized since the 1950s as more broiler chickens were grown in large numbers for commercial production [76]. Sudden death syndrome (SDS) and ascites together can account for 50% of the mortality of highly productive broiler chicken strains [85,86].

The parent generation of broiler chickens grown for meat shares the same genetics as their progeny. Like breeding sows, these broiler breeders must be feed restricted, or they will suffer from health and reproductive disorders due to weight gain. Parent birds are usually feed-restricted starting when they are as young as one week old [87]. In many parts of the world broiler breeders may be fed on a “skip-a-day” regimen, in which the animals are fed as infrequently as every other day—though this practice has been outlawed in several European countries [88]. Experimental studies suggest that artificial selection for increased body weight may have altered the brain mechanism controlling satiety and appetite [89], and evidence from behavioral studies suggests that feed restriction interferes with learning [90] and causes stress [91,92], boredom, and chronic hunger [93,94,95]. Breeding birds receive only 25%–50% of the amount of feed they would otherwise eat if given free access [93]. While free-range chickens normally devote about 50% of their daily time budget to foraging [96,97], feed restricted parent birds can consume their daily feed allotment in as little as 15 min [87]. Feed restriction is believed to cause undernourishment, nutritional deficiency, and abnormal behavior including increased pecking at non-feed objects, pacing, and heightened aggression [87,98]. Given that target body weights for broiler breeders have changed little in the past 30 years, but broiler body weight continues to increase, the welfare of parent birds may become more serious in the future as they will be feed deprived to an even greater extent [99].

#### 2.2.4. Genetic Selection with Regard to Welfare

While genetic selection for productivity can have negative side-effects for animal welfare, this is not necessarily always the case. Although progress may be slow, it is possible to select for both welfare associated traits and productivity simultaneously. For example, while there is an overall negative correlation between walking ability and growth rate of broiler chickens, some individuals do have both good gait scores and high productivity. Given enough time, it might be possible to successfully select for both traits [100]. Where animals can be bred to produce the same amount of meat or milk, but with less feed, GHG emission intensity (CH_4_/unit animal product) is reduced. The most efficient animals produce less CH_4_ and N_2_O than the population average [101], and there might not be a further welfare impact if selection focused on these animals. Direct selection on reduced CH_4_ production could also be a possibility. For example, in one study of sheep the heritability of g CH_4_/day was 0.29 with no genetic correlation with live weight, and a slightly economically favorable correlation with fleece weight [102].

#### 2.2.5. Mismatching Genetics and Environment

According to the FAO, the single most effective GHG mitigation strategy in many regions of the world is to increase animal productivity [10] (p. 100). Raising imported animals selected for production traits, instead of traditional breeds is part of that strategy. However, the FAO also cautions that while achieving the genetic potential of highly productive breeds is important, these animals should not be imported into environments where climate or management limitations would prevent the animals from achieving this potential. In new regions or countries, animals from genetic lines with high production potential may actually perform worse than native breeds or crossbreeds, especially compared to those well-adapted to local disease and climactic conditions [10] (p. 104). Some regions have seasonal fluctuations in feed quality and nutrient management, and, as high genetic merit breeds have exacting nutritional requirements, they do not always thrive as expected.

In southern India, for example, efforts to replace the native Vechur cow with imported Holsteins have been disappointing, because Holsteins were not bred for performance under the local conditions. While the small Vechur cows produce only one-tenth of the milk hoped for from Holsteins, they are hardy, heat and humidity tolerant and resistant to local diseases and ticks. Under the these environmental conditions in India, Holsteins do not produce the same milk yield that they do in more temperate climates, and the costs of keeping the animals alive and healthy may be greater than any added productivity [103].

In Africa, resistance to trypanosomiasis is a factor that limits the geographical distribution of cattle breeds. Indigenous taurine (humpless) breeds of cattle including the N'Dama and West African Shorthorn are genetically trypanotolerant [104], but European taurine breeds have no resistance, and even Zebu cattle imported from other regions of the continent do not thrive in the humid tropics of West Africa occupied by tsetse flies, vectors of trypanosomiasis [103]. Clearly there are limits to importing more highly productive breeds in this large swath of the world, and such circumstances call in to question the notion of “genetic improvement”—indeed the improved breeds here would appear to be the ones that can best survive the local conditions.

In Brazil, more fertile and adapted breeds, such as the Brazilian Gir, are increasing in popularity [105]. Indigenous breeds are better adapted to local conditions, and more readily able to avoid predators, tolerate heat or other weather extremes, resist parasites and endemic diseases, and scavenge for feed or consume local feed resources. For example, in the subtropical climate zone, with seasonal rainfall patterns that do not permit continuous vegetation growth, Zebu and Sanga cattle are more resistant to ticks and tick-transmitted diseases as well as feed scarcity and seasonal high temperatures [106].

While promoting highly productive genetics to address climate change will reduce GHG emissions in some ways, maintaining biodiversity and the genetic resources of locally adapted breeds will be needed to complement other climate change mitigation strategies and to ensure the animals thrive. A potential solution may be more research and genetic selection of local breeds, but only with due consideration to welfare. Cradle-to-grave analyses that include the full range of trade-offs will become increasingly necessary [107]. Genetic suitability for survival in less hospitable regions with more variable climates and diverse, changing feed resources is currently, and may become even more so, an essential requirement for both human and animal welfare.

### 2.3. Growth and Yield Promoting Biotechnologies

Some authors are calling attention to the wide application of growth promoting technologies, including β-adrenergic agonists (βAAs) and recombinant bovine somatotrophin (rbST), for their climate change mitigation potential [108,109]. While the research to date has focused on US production systems, the call is being made in the context of meeting increasing global demand for animal protein. Zilpaterol hydorchloride and ractopamine hydrochloride are βAAs that are used as feed additives in grain rations for cattle [110]. βAAs are fed in a 20–40 day period prior to slaughter to improve feed efficiency and weight gain [111]. The growth hormone rbST is used in the dairy industry to increase milk yield and feed conversion efficiency [108]. However, both rbST and βAAs are cause for substantial animal welfare concern [110,112,113].

#### 2.3.1. β-Adrenergic Agonists

Using Life Cycle Analysis (LCA) modeling, Stackhouse *et al.* (2012) found that the use of βAAs, in combination with antibiotics, ionophores, and hormone implants, as is common in US feedlots, decreased the carbon footprint of Angus beef production by 9% [109], and therefore advocated their use as a potential mitigation option. Cooprider *et al.* (2011) calculated that feedlot cattle fed ractopamine hydrochloride along with hormone implants and antibiotics had a 31% decrease in emissions per finished steer given the improved feed efficiency [114]. However, anecdotal reports of changes in behavior and more cattle deaths when βAAs were being fed has led researchers to investigate [109]. One recent study found that cattle fed βAAs along with a growth implant (trenbolone acetate and estradiol) engaged in more agonistic behavior including bulling and pushing [115]. A large-scale investigation by two major US agricultural universities examined datasets provided by feedlot operators also confirmed the field observations, finding that the cumulative death rate is as much as 90% greater in animals administered a βAA, and that 40%–50% of deaths among groups administered a βAA could be explained by administration of the drug [110]. Imports of animal products containing beta agonists is banned in many countries, including the EU, Russia, and China [116].

The βAA ractopamine hydrochloride is also fed to swine, where the side-effects that have been elucidated to date include elevated heart rate, difficulty walking [117], increased aggression [118], abnormal behavior [119], and hoof lesions [120]. Observations at slaughter plants additionally find that difficulty walking due to ractopamine may contribute to a greater incidence of non-ambulatory (or “downed”) pigs, those too weak to stand and walk on their own accord [117]. The number of studies investigating the animal welfare effects of βAAs is, thus far, small, but given the problems identified already, calls for their increased use clearly should be made more cautiously.

#### 2.3.2. Recombinant Bovine Somatotrophin

Also called Bovine Growth Hormone, rbST is a protein hormone that redirects nutrient partitioning during lactation and can increase milk yield by 8%–36% [121]. On a per unit of milk produced basis, it has been show that rbST reduces the maintenance energy and protein requirements of cows by 11.8% and 7.5%, respectively, and total feed requirements by 8.1% [121]. It also reduces N excretion on a per liter of milk produced basis. Although overall N excretion increases in rbST treated cows, because they consume more feed, each cow produces more milk, so estimates calculated on a per liter basis report a reduction in nitrogen excretion between approximately 9% [108] and 15% [122]. Per unit of milk, the dilution of maintenance conferred by the use of rbST can result in a reduction in manure production by 6.8% and CH_4_ output by 7.3% [108].

While rbST is banned in the European Union, Canada, Japan, Australia, and New Zealand [10], the drug is approved in the United States, despite substantial controversy. One of the primary welfare concerns associated with the increased milk production caused by rbST is the potential risk for mastitis. Some of the initial studies on rbST indicated that its use was linked to an increase (for example, Burton *et al*., 1990 [123] and Pell *et al*., 1992 [124], but see White *et al*., 1994 [53] for counter examples).

A 1999 report of the Scientific Committee on Animal Health and Animal Welfare (SCAHAW), a working party of independent scientists appointed by the European Commission to assess rbST, reviewed approximately two dozen studies and concluded that rbST usage increases the risk of clinical mastitis and the length of infections [113]. This finding was supported by a review published by the Canadian Veterinary Medical Association (CVMA) [112]. The CVMA performed a series of meta-analyses to combine data from all randomized clinical trials that had been published in peer-reviewed journals or which were provided by Health Canada for the registration of rbST in the country. The CVMA found that rbST increased the risk of clinical mastitis by approximately 25%, closely agreeing with the SCAHAW report. Subsequently, rbST was not approved for use in Canada, due to animal health concerns.

The debate between researchers in different countries largely centers on the question of whether increases in mastitis are due to rbST treatment *per se* or to the increase in productivity caused by rbST, the same as would be expected when genetic selection leads to greater milk production. White *et al.* (1994) argue that treatment differences in mastitis are within the range predicted using estimates from genetic studies. And using this logic they conclude that rbST treatment had no effect on mastitis [53]. In contrast, SCAHAW (1999) argue that what matters is the result on the welfare of the animals, which is the same whether it is the drug itself or the increase in productivity that causes the mastitis [113].

In addition to problems with mastitis, there is also concern that rbST treatment may increase the risk of lameness and foot and leg disorders. While many early studies failed to find a detectable difference between rbST treated and control animals [121,125,126,127,128,129], others found a slight increase in the number of cows culled due to lameness [130] and slightly more feet and leg problems [130,131]. As with the early work on mastitis and rbST, in studies with small cow numbers, low statistical power may have prevented detection of effects, and slight increases would be hard to find.

However, both the SCAHAW committee and the CVMA review panel found an increased incidence of foot and leg disorders associated with the long-term administration of rbST [112,113]. The CVMA review panel found that cows treated with rbST were at an estimated 55% increase in the risk of developing clinical signs of lameness, and this is now reflected on the Posilac drug label, which states “Cows injected with Posilac may have more enlarged hocks and disorders of the foot region” [132].

Recombinant bovine Somatotropin has also been shown to reduce the ability of cows to cope with high environmental temperatures, which can lead to heat stress [113,133]. It is, thus, not advisable in geographic regions where severe heat can be expected, especially if management options to alleviate heat stress are insufficient.

Clearly the use of rbST carries unintended risks to the welfare of animals, and these should be seriously considered before such growth-promoting compounds are touted as a possible mitigation strategy for climate change.

### 2.4. Species Shifts

In addition to increasing productivity within species, the current global trend toward intensively producing greater numbers of non-ruminant animals—pigs and chickens—is often viewed as a more efficient form of meat production compared to traditional ruminant cattle, sheep and goat rearing systems [134,135,136]. To meet the growing demand for animal protein in emerging economies [13], the expansion in supply is largely coming from increased local production, as opposed to from imports [12], and in these regions, livestock systems are rapidly shifting toward monogastric species. Pigs and poultry account for 77% of the expansion in production, a trend that is often accompanied by intensification, vertical integration, geographic concentration, up-scaling of production, and feeding more grain- or concentrate-based diets [12]—in short, industrialized farming.

Monogastrics have improved GHG efficiencies compared to cattle. Cattle accounted for 77% of total non-CO_2_ GHG emissions from the livestock sector in 2000, while monogastrics accounted for just 10% [136]. It takes at least three times as much land to produce one kilogram of beef as it does to produce one kilogram of pork or chicken [137]. Cattle contribute about seven times the CO_2_-eq. emissions of pigs and chickens [2]. This biological difference can be explained by three main factors: greater feed efficiency, lower enteric CH_4_ emissions, and faster rates of reproduction. CH_4_ emissions from ruminants originate from both manure and enteric fermentation, while CH_4_ emissions from monogastrics are primarily associated with manure alone. Because sows and hens have a relatively large number of progeny and earlier sexual maturity, the number of breeding adults per animal slaughtered for human food is reduced in these production systems [137]. Together, these factors account for the reduction in inputs and lower emissions of pig and poultry production worldwide.

If the increasing consumption of animal protein in developing countries will be supplied in a substantial way by expansion of pork and chicken industries, and if the expansion will be in the form of specialized, intensive confinement facilities that typify the bulk of production in North America and other developed regions, then there is a serious potential for a concomitant increase in the animal welfare problems that characterize those systems.

As covered in Section 2.2.3, the intensive production of modern strains of poultry is the cause of one of the most severe and wide-spread animal welfare problems in the world. Globally, nearly 60 billion chickens are slaughtered for meat each year [138], and given the wide prevalence of metabolic problems and painful leg disorders, the intensive production of meat chickens already constitutes one of the most problematic aspects of intensive agriculture. Sweeping calls to use *more* of these compromised animals to meet efficiency goals is alarming.

In an ethical analysis, overall welfare would be a function of both the state of the individual animal and the number of animals subjected to that level of welfare. It takes far more animals to produce the same amount of meat when beef is replaced in the diet with chicken, compounding the overall magnitude of any negative animal welfare impacts.

Another of the most salient issues with intensive confinement of animals in agriculture is the way those animals are housed. Intensive systems for pork and poultry production remove animals from the land and instead raise them in indoor, total confinement facilities. While well-managed, large-scale production can have some animal welfare advantages, such as access to resources that might be economically out of reach to smaller producers, they often greatly limit the space allotment per animal. In the case of breeding sows in the pork production industry the animals are further partitioned into enclosures that prevent normal movement and severely restrict the animals’ natural behavior, which is a serious welfare problem.

#### 2.4.1. Industrial Production of Pigs

Swine are highly intelligent, social animals with complex behavioral repertoires. Yet, in many countries, intensive production facilities house female breeding pigs in narrow gestation crates, metal stalls that measure approximately 0.61 m by 2.31 m, which is only slightly larger than the sow’s own body. Each sow can take a step forward and backward, but she cannot turn around for the length of her pregnancy, which is approximately 114 days. Due to decreased fitness from chronic lack of exercise, crated sows have lower bone strength and muscle weight [139], and have higher resting heart rates [140], compared to those permitted more freedom of movement and exercise.

Fed concentrated grain-based diets that can be consumed quickly, and without opportunity to express species-typical foraging behavior, sows in intensive confinement operations often begin to display abnormal, stereotypic behavior including repetitive bar-biting, head-weaving, sham-chewing and drinker pressing. Rooted in stress and lack of stimulation [141,142], these “stereotypies”, are common in captive animals kept in barren, restrictive environments [143]. Stereotypic behavior is often reduced when sows are fed a high fiber, bulky diet instead of, or in addition to, concentrates [144,145,146,147,148], although some fiber sources, such as soybean hulls, may be less effective [149]. Ethologists attribute this stereotypic behavior to boredom and frustration resulting from an impoverished environment, confinement, restraint, and unfulfilled behavioral needs [150,151].

Most large, industrialized confinement facilities for pigs utilize a liquid manure handling system with slatted floors. While a 2013 FAO review noted that slatted floor manure systems tend to decrease GHG and NH_3_ emissions compared with deep litter systems [10], these bare, concrete floors are hard on the animals’ feet. One survey found that pigs allowed outdoor access had a lower prevalence of foot and limb injuries, while those confined indoors on hard, slatted flooring had more bruising, calluses, locomotion problems, and adventitious bursae [152]. The problem is especially acute for piglets; foot and limb injuries affect 60% to 90% of young piglets housed indoors, while only about 9% of piglets housed outdoors suffer these types of injuries. While outdoor production does have its own suite of welfare concerns, including severe weather and predators, in farrowing huts common to pasture-based systems, soil covered with straw provides a soft, non-abrasive, protective surface [152]. Behavioral testing has shown that pigs prefer earthen floors over concrete [153,154]. While bedding, such as straw provides comfort, warmth and outlets for natural rooting and nesting behavior, it is not usually provided in indoor operations due to cost, difficulty of cleaning, and incompatibility with slatted floors.

There are however, more welfare-friendly intensive systems. For example, sows can successfully be housed in group pens as opposed to individual gestation crates, as long as they are managed well to prevent aggression. Group housing systems work best if they deliver the feed in a non-competitive way, such as by using trickle feeding, feeding stalls, or Electronic Sow Feeding systems (which use a computer to precisely deliver each sow’s daily allotment) [155]. Straw bedding can be incorporated into these systems in purpose-built un-slatted sections of the barn. However, N_2_O emissions in a straw-based, deep litter group housing systems are usually greater compared to concrete slatted floors [156]. Further research on group housing systems design could focus on lowering emissions while simultaneously fulfilling the behavioral needs of the animals.

#### 2.4.2. Further Disadvantages of Shifting Production Systems

Another problem with large, industrialized animal production sites is the ratio of animals to human caretakers. With increased farm size, new technologies and increased mechanization, such as automated feed and water delivery and manure removal, coupled with economic pressure to reduce labor, the result is that fewer workers now tend to more animals. As such, individualized attention to each animal is generally lacking. With the use of efficiencies in pen and barn design, one person may now be responsible for the care of 8000 pigs per day [157] (p. 291). One of the main daily tasks on a commercial-scale broiler grow-out facility is picking up the dead birds. While large farms can be well managed to reduce death losses, the health of the animals on large operations is often addressed at the herd or flock level, and individual veterinary care is not generally provided. An individual animal who becomes injured or ill is likely to go unnoticed, and could experience prolonged pain and suffering before their eventual death.

On top of the animal welfare concerns, the industrialization of agriculture carries with it numerous other societal implications. Switching to large-scale use of monogastrics threatens genetic diversity, and subsequent genetic adaptation of domestic species to climate change [158]. It could also mean the proliferation of more and more serious environmental issues, including air, soil, and water pollution. For example, the rapid expansion of pig production in the coastal areas of China, Vietnam, and Thailand is thought to be a major source of nutrient pollution in the South China Sea. Runoff is causing red tides and threatening fragile mangroves and coral reefs [12] (p. 71). Concerns such as these must be properly weighed in the discussion of intensification and shifts to monogastric production as proposed climate change mitigation strategies.

System changes, however, (e.g., from extensive to industrialized production) are not required for some of the most substantial emissions reductions, which can be achieved within production systems, across all species and regions. The FAO found that applying the techniques of 10% of the most efficient producers to current systems, by for example, improved pasture management and feed quality, and better animal health, among others could cut nearly a third of emissions from the farm animal sector. Production system changes further reduced emissions a mere 2% [2] (pp. 45–46). Clearly, there is a large opportunity to improve emissions by picking the low-hanging fruit, *i.e.*, the least efficient producers [2,159], while achieving multiple benefits, such as animal welfare, or at least avoiding additional negative impacts. Such improvements should also aim at improved food security (not just increased production) and fit well within the concept of “sustainable intensification” [160].

## 3. Potential Solutions: Mitigation Strategies that both Improve Animal Welfare and Reduce Environmental Impacts

While it is clear that animal welfare concerns for many different climate change mitigation strategies exist, fortunately a great many more potential solutions have benign or even beneficial welfare outcomes. Many of these options are strategies for extensive production, systems that, if improved, have very high welfare potential. Basic improvements to these systems would also have the co-benefit of improving the livelihoods of smallholder farmers. With more attention paid to the health, physiology, and behavioral effects on animals at the outset, triple-win strategies can be championed that mitigate climate change, improve food security, and improve animal welfare.

### 3.1. Improved Animal Health and Longevity

Where climate change solutions and improved animal welfare may dovetail best begins with a focus on the animals. Improving the efficiency of animal production systems by improving animal health and reducing mortality could reduce CH_4_ and N_2_O emissions considerably by reducing losses due to health related production declines and emissions attributed to animals that die before they can reproduce or produce consumable products. In locations where basic veterinary care, vaccinations, clean drinking water, shade, husbandry skills, technical knowledge, financial aid and other vital resources are lacking, there is great potential for improvement. For example, in many developing African and Asian countries, approximately 80% of poultry is still located in village flocks [161]. Globally, backyard flocks kept for meat and eggs contribute 42.5 million tons of CO_2_-eq. in greenhouse gas emissions and have higher emission intensity than intensively confined chickens, due in part to the low feed quality of scavenging birds and the toll that disease and predation take on productivity [2]. Vaccinations, providing simple, low-cost protection for newly hatched chicks from predators, supplementary feeding of balanced rations, and basic biosecurity education [162] could simultaneously improve the welfare of the flocks, alleviate poverty, and potentially reduce greenhouse gas emissions. The climate change mitigation potential of these improvements has not specifically been studied, to our knowledge.

Extending dairy cow lifetime is another potential improvement. Cows can easily live to 15 years of age or longer, but on most intensive, indoor dairy production facilities that typify developed countries the lifespan of a cow is typically closer to six years [163]. A USDA summary reports the proportion of dairy cows permanently removed from the herd for reproductive problems was 26.3%; 23.0% were removed for udder or mastitis problems, 16.0% for lameness or injury, and 16.1% for poor production [52]. Similar factors for culling are reported for Canada and European countries [163]. In one study of 14 Irish dairy herds, the rate at which cows were replaced in the herd increased from 16% in 1990 to 27% in 2003, an increase of 0.8% per year, and the proportion of cows culled for infertility increased 2.2% per year, from 27% in 1990 to 40% in 2002 [164]. Improved longevity would reduce the total lifetime emissions of dairy cows when accounting for the resources needed for rearing replacement heifers. The proportion of total methane emissions produced by replacement heifers has been estimated to be up to 27% for U.K. dairy herds [165]. Relative to the period from birth to first calving (a period that usually lasts from 25 months to about 3 years, depending on the region), a greater number of lactation periods per lifetime will reduce overall CH_4_ loss per unit of milk yield [166]. By improving the lifespan of a dairy cow from 3.02 to 3.5 lactations, methane emissions would be reduced by 3% [167]. Selection for increased productive life could also lead to a decrease in disease incidence of dairy cows [168].

In the future, one possibility for addressing the lack of individual attention on industrialized farms could be “Precision Livestock farming” (PLF). In PLF, sensors continuously monitor key indicators, such as animal movement, feed intake, or temperature and alert caretakers when a problem arises. Examples include using cameras to monitor the behavior of hens, pedometers to monitor estrus behavior of dairy cows, and automated scales for measuring weight gain of chickens [169]. PLF has good potential for automated detection of animal health problems. For example, auditory sensors can be used to detect disease in fattening pigs by analysis of coughing sounds, which change qualitatively when the respiratory system is infected [170,171]. However, it is uncertain how applicable these highly technical solutions would be in developing countries, and there is a danger of losing genuine husbandry skills and knowledge when relying even more heavily on automation.

### 3.2. Improved Animal Nutrition

As discussed previously, changes to animal diets can reduce greenhouse gas emissions and increase animal productivity, but they can also risk harming animal welfare. However, bolstering nutrition by supplementing poor diets or using improved forages can both reduce greenhouse gas emissions and improve welfare at the same time. In regions of the world with a seasonally dry tropical climate, such as Africa, the low nutritional value of most animal feeds during the dry season is a major constraint on animal productivity [172]. In developing countries, ruminant animals are often fed on low quality grasses and crop residues (stover). Inadequate nutrition is a key factor limiting fertility [10]. A large potential to mitigate emissions exists in these low-yield systems. For example, modeling of small ruminant production in West Africa suggests that improvements in forage digestibility, along with other animal health and husbandry measures, can potentially reduce emissions by 27% to 41% of baseline emissions, amounting to 7.7 to 12 million tons of CO_2_-eq. [2].

Improved forages can be used as a climate change mitigation strategy in both developing and developed regions. Legumes available for grazing ruminants in the United States include clover, alfalfa, crown vetch, and birdsfoot trefoil (lotus). These nutritionally valuable, high protein forages can be incorporated into established pasture or rangeland with interseeding. Not only can improved pastures reduce CH_4_ emissions from animals, but carbon sequestration in grassland soils can also be increased by planting nitrogen-fixing legumes [173,174,175].

The mitigation potential of feeding legumes has been the subject of a substantial number of studies. Simulation results in one study showed that CH_4_ production is 28% lower with legume forage (alfalfa hay) as compared to grass (timothy hay) when expressed as a proportion of gross energy (GE) intake, and 21% lower when expressed relative to digestible energy (DE) [176]. These results are corroborated by multiple field studies, which also find lower CH_4_ with pasture legumes for sheep [177] and cattle [178,179]. For example, Waghorn *et al.* (2002) found that when fed to sheep, a sulla/lucerne feed mixture produced just 19.0 g CH_4_ per kg DMI compared to fresh pasture, which produced 25.7 g CH_4_ per kg DMI. In a second trial, lotus produced just 11.5 g CH_4_ per kg DMI, and by using polyethylene glycol binding to remove condensed tannins (CT), the researchers demonstrated that CT played an important role in this reduction [177]. Condensed tannins not only lower CH_4_ production in sheep, but may also have other beneficial effects including improving wool growth and reducing intestinal worm burdens [180]. However, at least one study failed to find reduced methane production feeding white clover to sheep (Hammond *et al.*, 2011) [181], and so more study is required.

One caution: legume consumption in some cases is associated with animal health issues, such as fetal hydrops in periparturient ewes [182], and bloat with some tropical legumes [183]. Feeding legumes must, therefore, be carefully managed to avoid animal health problems.

Many other feeding management strategies that can reduce greenhouse gas emissions without compromising animal welfare could be better emphasized and further explored. These include improving silage quality with more effective preservation methods [10]; adding dietary fats, such as coconut, linseed, soybean, or sunflower seed oils [184,185,186]; changes to pasture management such as matching grazing intensity and pasture quality [173,187] supplementation of the diet with enzymes [122,188] or yeasts [189], and precision feeding (better agreement between the supply of N in the diet and requirements of the animal, in order to reduce N excretion) [190,191], to name just a few.

### 3.3. Improved Manure and Land Management

In addition to improving animal health, nutrition or longevity, a great many more climate change mitigation options remain that could be used without negatively affecting the welfare of animals. Ruminants may be key parts to well-managed, mixed crop-livestock systems, helping to reduce a number of environmental impacts [192]. Well-managed grazing can improve soil organic carbon and nitrogen content [193] and partially offset net GHG emissions [194]. Most manure management options are probably welfare-neutral, unless collection systems require the intensive confinement of animals. Hristov *et al.* (2013) provide a thorough summary of such options including reducing manure storage time, composting, and carefully timed land application [10]. Several techniques for manure application also hold mitigation promise, for example subsurface application, application during non-rainy periods, and controlling soil pH. Perennial crops can be used to improve soil structure, water retention capacity and, in turn, carbon sequestration capacity [195]. Converting from conventional tilling to conservation or no-till cropping practices, especially with the simultaneous use of cover crops, can reduce soil disturbance and improve soil biodiversity and is another way to sequester carbon [196]. Other options include restoration of degraded range land (re-vegetation, water conservation) [197,198], and silvopastoral and agroforestry systems [198,199] among many others.

### 3.4. Reduced Animal Numbers

The demand for meat and dairy products is rapidly growing. This demand is attributable to the growing world population, urbanization and increasing affluence in emerging economies [200]. The projected global growth in annual consumption of meat and milk is 5.41% and 3.43%, respectively. In Asia this growth is projected to be even greater, at 7.99% and 11.85% [201]. Meat consumption is expected to continue to grow in developing countries until they meet the levels of developed countries, with poultry accounting for around 50% of the increase [202]. If this demand is met, the growing number of animals will substantially increase the problem of climate change [203], even if production efficiencies per unit of product produced are realized.

The growing demand for animal-based protein is usually presented as inevitable. While the general trajectory seems certain, the call to fill this demand in every case is not necessarily prudent. Reducing animal numbers could come, in part, with changes in production efficiency, as mentioned previously [2]. However, these production-side efficiency approaches seem, in some ways, to avoid the heart of the problem.

Feeding animals grain to produce animal-based food is inherently inefficient. For every kg of chicken live weight gain, 1.7 kg is required in feed, and chicken is the most highly feed efficient species; the ratio of feed to gain for pigs is 2.4 to 1 and for cattle it is as high as 10 to 1 [204]. The production of feed for animals can have a larger environmental impact in terms of energy use and global warming, than the management of those same animals and their manure on the farm [205,206]. Using IPCC figures, Garnett (2011) estimates that the reductions achievable for agriculture mitigation options amount to only about 30% of the total impact [207]; following this she argues: “[h]ence the conclusion that we need also to moderate our consumption of livestock products seems inescapable.”

One approach to this is to address the immense amount of food that is wasted. This category is broad, including waste at harvest, storage, processing, and a number of post-consumer pathways [208]. The inefficiencies above suggest that waste of animal-based foods carries a particularly high environmental cost. The FAO has estimated global food waste at about 1/3, with waste of meat products of about 20% [209]. Smith *et al.* (2013) found savings of about 6% in food crop area, and 13%–47% GHG savings in 2050 through waste reduction compared to the reference case [210].

Another approach focuses specifically on consumption, particularly in developed countries and in populations that face chronic disease due to overconsumption. Many studies show that reducing consumption of animal products would have a substantial effect on reducing GHG emissions, and the IPCC recognized this potential in their Fifth Assessment Report [4]. Smith *et al.* (2013) found greater mitigation potential in the Agriculture, Forestry, and Other Land Use (AFOLU) sector on the consumption rather than production side, when incorporating associated benefits in spared land use (e.g., afforestation and bioenergy) [210].

While variables such as energy used in transport, processing and refrigeration do not permit blanket statements about animal products over alternatives, meat, seafood, and some dairy products tend to have large GHG emissions per kg of product at the checkout as compared to most plant-based foods [211]. Some analyses show that vegetarian and vegan diets have potential GHG savings of 22% and 29% in the UK and US, respectively [212,213]. However, a 2014 study conducted in the UK found that dietary GHG emissions for meat-eaters were approximately twice as high as those of vegans [214].

Vegan and vegetarian diets also have lower environmental impact including less land use [215], water [216], and fossil fuel requirements [217]. Reducing climate change mitigation costs [215], as well as potential to improve public health [218], have also been cited as a co-beneficial effects.

Substantial effects could be realized even by simply reducing meat consumption, such as through efforts like the Meatless Monday campaign [219], or partial substitution of plant protein in ground and processed meats [200]. Another potential future alternative is cultured meat production. Cultured meat is laboratory produced using *in vitro* tissue engineering techniques, and initial LCA estimates show that it could lead to 78%–96% lower GHG emissions than conventionally produced European meat [220], while simultaneously avoiding all of the animal welfare impacts of intensive production.

## 4. Conclusions

Proposals for climate change mitigation strategies often use the term, “sustainable intensification.” Yet, societal norms related to the way we treat animals and use them in agriculture are changing and some past practices are no longer as widely accepted. Intensification of animal farming may not ever truly be sustainable, unless, among other things, there is concomitant attention to the health and behavioral needs of the animals, a meaningful effort to provide them with a life worth living. There is an urgent need to integrate these other sustainability measures into GHG mitigation assessments [221].

While multiple approaches are necessary to help stop anthropogenic climate change, those that offer the largest potential climate and sustainability benefits, while avoiding negative tradeoffs, should be the focus. This could mean different approaches (and levels of focus) for pre-market (production side) *versus* the market and beyond (demand side). For example, with production-side measures, there can be a rapid marginalization of further climate benefits beyond initial productivity gains [159]. Demand-side approaches, on the other hand, seem to offer a largely untapped potential with multiple co-benefits.

More research is needed to explore the combination of animal welfare and environmental improvements. There are some promising examples in pigs [222], chickens [223], and dairy cows [224,225]. These studies exhibit potential and the factors dictating these results, as well as ways to improve upon them, should be the focus of future research agendas.

Calls to globalize the production systems that were developed in the West are concerning when the impacts on the animals are taken into account, and there is worldwide momentum toward abandoning the worst practices. For example, multinational corporations such as McDonalds, Compass Group, and Nestle are asking producers for plans to eliminate gestation crates from their supply chains. Legislative and voluntary phase-outs of intensive animal confinement systems are increasingly taking hold across the globe, for example in the European Union, South Africa, Australia, Canada, New Zealand and several US states, with active campaigns in many additional regions of the world. The global movement toward farm animal welfare reform must be an important consideration going forward in the discussion of climate change mitigation strategies, and research and policy should increasingly reflect this.
